# Perceptions of Occupational Risk and Adherence to Tuberculosis Prevention Among Health Care Workers: Protocol for a Scoping Review

**DOI:** 10.2196/64037

**Published:** 2025-09-22

**Authors:** Agus Fitriangga, Alex Alex, Eka Ardiani Putri

**Affiliations:** 1 Department of Community Medicine Faculty of Medicine Tanjungpura University Pontianak Indonesia

**Keywords:** health care worker, tuberculosis, occupational risk, perceptions, TB prevention

## Abstract

**Background:**

Tuberculosis (TB) is a major public health problem around the world. Health care workers (HCWs) are at a much higher risk of contracting TB because they are often working around sick people in clinical settings. Even though HCWs play a key role in controlling TB, we still do not fully understand how they see this risk and how it affects their willingness to follow preventive measures.

**Objective:**

This study aims to examine the existing body of knowledge on HCWs’ perceived risks of TB and how these perceptions impact their adherence to TB prevention measures. The results of this scoping review will identify gaps in the current literature that should inform policy and practice and guide future research studies to optimize TB prevention among HCWs.

**Methods:**

This scoping review will be conducted following the framework proposed by Arksey and O’Malley, incorporating the recent advancements. This approach involves 6 key stages: identifying the research question; identifying relevant studies; selecting studies; charting the data; collating, summarizing, and reporting the results; and consulting with stakeholders.

**Results:**

As of June 2024, 1345 records were identified (1234 from databases and 111 from other sources), and 667 duplicates were removed. The remaining 678 records were screened by title and abstract, with 216 progressing to full-text review. After applying the eligibility criteria, 42 studies were included in the final analysis. Screening and full-text assessments were conducted between September and October 2024. Data extraction and thematic analysis were performed in winter 2024. The final data synthesis stage is expected to be completed by 2025.

**Conclusions:**

HCWs’ perceptions of risk have a considerable effect on how well they follow TB prevention measures such as using personal protective equipment and undergoing health screenings. Lack of resources, lack of training, and the stigma around TB are some of the main barriers to TB prevention adherence. The thematic analysis showed that adherence levels were different depending on the support offered by the institution and the TB knowledge level and perception of each HCW. Although TB treatment has become more effective, nosocomial infections are still a big concern, especially in low- and middle-income countries like Indonesia, where HCWs are more likely to have latent TB infections. This review shows how important it is for HCWs to understand how TB prevention behaviors work. To improve HCW adherence, the gaps in institutional support, stigma, and training must be filled. Future interventions should be based on the specific problems found in low- and middle-income countries. This will make health care safer for everyone around the world.

**International Registered Report Identifier (IRRID):**

DERR1-10.2196/64037

## Introduction

Tuberculosis (TB) remains a major global public health issue and a particular concern among health care staff in the high-risk environment of clinical settings. Health care workers (HCWs) are at the forefront of TB control and consequently to occupational *Mycobacterium tuberculosis* exposure. Compared to the general population, HCWs are at a higher risk of TB infection, as the dependent factor of the World Health Organization (WHO) has estimated HCWs to be approximately 2-3 times more likely to become infected with TB. Earlier landmark research published in 2001 had provided evidence that TB can be transmitted through air in health care environments [1-3]. Thus, we need control measures to reduce this risk.

TB has the largest impact in low- and middle-income countries (LMICs), as >95% of TB-related deaths occur in LMICs. Only 8 LMICs account for two-thirds of the global caseload, and one of them is Indonesia [4,5]. The risk of HCWs developing TB is much higher than that of the general population [6-8]. A systematic review of 111 studies showed that the prevalence of latent TB infections (determined by the tuberculin skin test) among HCWs in LMICs ranged from 14% to 98%, with the mean prevalence being 49% [9,10]. High regional variability has been observed in the annual incidence of latent TB infections among HCWs, ranging from 0.5% to 14.3% [11,12].

Occupational risk perceptions can be compromised [13,14], contributing to nonadherence to proven effective TB prevention regimens (eg, personal protective equipment, regular enrollment in health screenings). Although there has been a notable advancement in the treatment of TB, nosocomial or hospital-acquired TB infections remain a considerable occupational risk, particularly with the complex interplay of multidrug-resistant TB and HIV/AIDS in clinical settings [15,16]. Studies have shown that HCWs’ TB preventive behaviors widely differ and are determined by their risk perceptions, knowledge levels, and institutional support. For example, HCWs at a higher risk of TB are generally more engaged in preventive behaviors [17,18]. Yet, persistent barriers remain, such as insufficient personal protective equipment, poor ventilation, and TB stigma [19,20]. Such gaps warrant strategic mapping of existing literature on HCWs’ perceptions and practices to inform future interventions. Our objective is to synthesize existing literature on HCWs’ perceptions of occupational risk and adherence to TB preventive routine measures in order to identify widespread themes and gaps in the research and provide an overview of the factors informing these perceptions and behaviors. This study aims to examine the existing body of knowledge on HCWs’ perceived risks of TB and how these perceptions impact their adherence to TB prevention measures. The results of this scoping review will identify gaps in the current literature that should inform policy and practice and guide future research studies to optimize TB prevention among HCWs.

## Methods

### Study Design

This study is a scoping review protocol and does not involve a randomized controlled trial; therefore, trial registration is not applicable. This review will be conducted following established methodological frameworks by Arksey and O’Malley [14], with enhancements by Levac et al [21], and guided by the Joanna Briggs Institute methodology for scoping reviews [22]. This approach involves 6 key stages: identifying the research question; identifying relevant studies; selecting studies; charting the data; collating, summarizing, and reporting results; and consulting with stakeholders. The PRISMA-ScR (Preferred Reporting Items for Systematic reviews and Meta-Analyses extension for Scoping Reviews) checklist is shown in Multimedia Appendix 1.

### Key Stages of the Study

#### Stage 1: Identifying the Research Question

The primary research question guiding this scoping review is as follows: how do HCWs perceive their occupational risk of TB and what factors influence their adherence to TB prevention and control measures? This question aims to explore the breadth of literature on HCWs’ risk perceptions and their impact on preventive behaviors.

#### Stage 2: Identifying Relevant Studies

**Figure 1:**
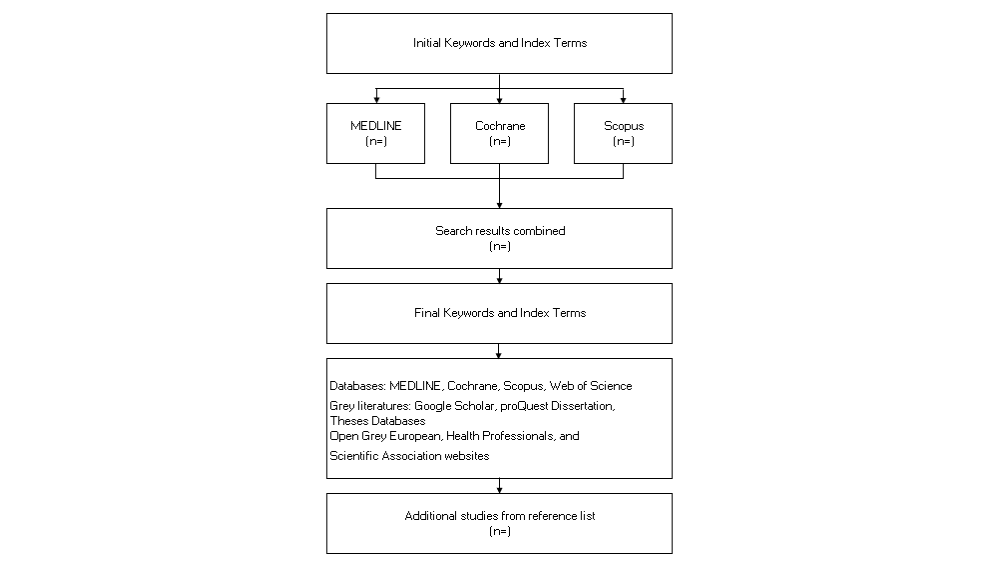
Search strategy.

The review will apply the Joanna Briggs Institute approach [23] to identify the suitability of the papers (Table 1). A comprehensive search strategy will be employed to identify relevant studies. Key databases, including PubMed, MEDLINE, EBSCO, Scopus, and Web of Science, will be searched. Keywords and search terms will include combinations of the following: HCWs, health personnel, tuberculosis, TB, occupational risk, perceptions, attitudes, awareness, knowledge, prevention, control measures, adherence, and compliance (Table 2). Both peer-reviewed studies and grey literature will be considered to ensure a wide scope of information (Figure 1).

**Table 1:** Search strategy for the perceptions of occupational risk and adherence to tuberculosis prevention among health care workers.

Strategy	Criteria
Population	HCWs^a^, including doctors, nurses, laboratory technicians, and support staff, directly involved in patient care Currently employed in health care settings such as hospitals, clinics, or health centers A minimum of 6 months of experience in a health care setting Willingness and ability to provide informed consent to participate in the study
Concept	Studies exploring HCWs’ perceptions of occupational tuberculosis risk and/or adherence to tuberculosis prevention and control measures
Context	Studies conducted in any health care setting (hospital, primary care, clinic) and in any country
Type of source	All research designs: observational studies, randomized controlled trials, systematic reviews, case studies, qualitative studies, clinical guidelines. The following were excluded: publications in the form of editorials, letters to the editor, comments, and case reports/narrative case reports.

^a^HCW: health care worker.

**Table 2:** Keywords and queries used for identifying the studies on the perceptions of occupational risk and adherence to tuberculosis prevention among health care workers.

Database	Keywords	Query
MEDLINE	Health care workers: health care personnel, health care professionals, medical staff, clinical staff, doctors, nurses, laboratory technicians Perceptions: perception, awareness, belief, attitude, understanding Occupational risk: occupational risk, workplace exposure, job hazard, professional risk Tuberculosis: tuberculosis, TB, Mycobacterium tuberculosis Prevention: prevention, infection control, protective measures, preventive practices, adherence, compliance	((“Health Personnel”[MeSH] OR health care workers OR medical staff OR clinical staff OR doctors OR nurses OR laboratory technicians) AND (“Perception”[MeSH] OR perception OR awareness OR belief OR attitude OR understanding) AND (“Occupational Exposure”[MeSH] OR occupational risk OR workplace exposure OR job hazard OR professional risk) AND (“Tuberculosis”[MeSH] OR tuberculosis OR TB OR Mycobacterium tuberculosis) AND (“Infection Control”[MeSH] OR prevention OR protective measures OR preventive practices OR adherence OR compliance))
Scopus	Health care workers: health care personnel, health care professionals, medical staff, clinical staff, doctors, nurses, laboratory technicians Perceptions: perception, awareness, belief, attitude, understanding Occupational risk: occupational risk, workplace exposure, job hazard, professional risk Tuberculosis: tuberculosis, TB, Mycobacterium tuberculosis Prevention: prevention, infection control, protective measures, preventive practices, adherence, compliance	KEY (“health care personnel” OR “health care professionals” OR “medical staff” OR “clinical staff” OR doctors OR nurses OR “laboratory technicians”)) AND (TITLE-ABS-KEY (perception OR awareness OR belief OR attitude OR understanding)) AND (KEY (“occupational risk” OR “workplace exposure” OR “job hazard” OR “professional risk”)) AND (KEY (“tuberculosis” OR TB OR “Mycobacterium tuberculosis”)) AND (TITLE-ABS-KEY (prevention OR “infection control” OR “protective measures” OR “preventive practices” OR adherence OR compliance))
EBSCO	Health care workers: health care personnel, health care professionals, medical staff, clinical staff, doctors, nurses, laboratory technicians Perceptions: perception, awareness, belief, attitude, understanding Occupational risk: occupational risk, workplace exposure, job hazard, professional risk Tuberculosis: tuberculosis, TB, Mycobacterium tuberculosis Prevention: prevention, infection control, protective measures, preventive practices, adherence, compliance	(“health care personnel” OR “health care professionals” OR “medical staff” OR “clinical staff” OR doctors OR nurses OR “laboratory technicians”) AND (perception OR awareness OR belief OR attitude OR understanding) AND (“occupational risk” OR “workplace exposure” OR “job hazard” OR “professional risk”) AND (“tuberculosis” OR TB OR “Mycobacterium tuberculosis”) AND (prevention OR “infection control” OR “protective measures” OR “preventive practices” OR adherence OR compliance))
Google Scholar	Health care workers: health care personnel, health care professionals, medical staff, clinical staff, doctors, nurses, laboratory technicians Perceptions: perception, awareness, belief, attitude, understanding Occupational risk: occupational risk, workplace exposure, job hazard, professional risk Tuberculosis: tuberculosis, TB, Mycobacterium tuberculosis Prevention: prevention, infection control, protective measures, preventive practices, adherence, compliance	“health care workers” OR “health care professionals” OR “medical staff” OR “clinical staff” OR doctors OR nurses OR “laboratory technicians” AND perception OR awareness OR belief OR attitude OR understanding AND “occupational risk” OR “workplace exposure” OR “job hazard” OR “professional risk” AND tuberculosis OR TB OR “Mycobacterium tuberculosis” AND prevention OR “infection control” OR “protective measures” OR “preventive practices” OR adherence OR compliance

#### Stage 3: Study Selection

**Figure 2:**
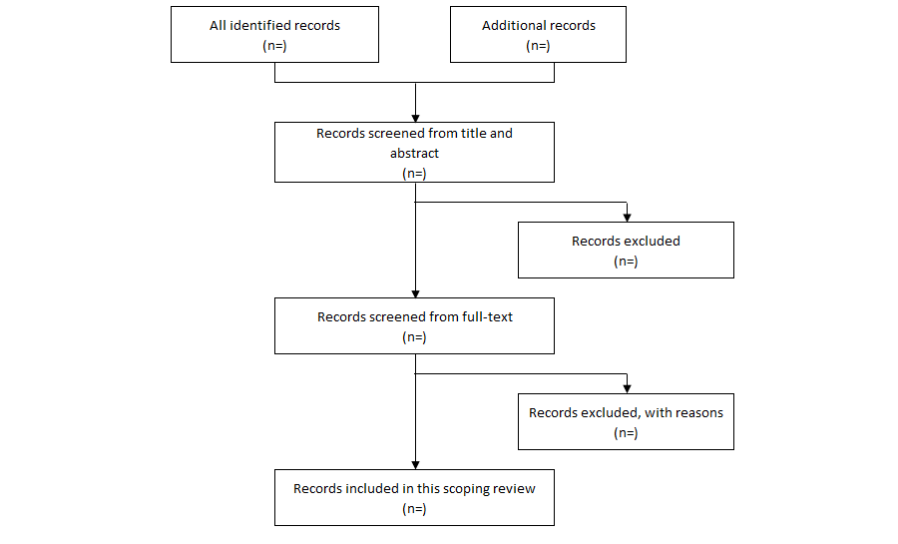
PRISMA-ScR flow diagram for the study selection.

The selection process will involve a 2-step approach. Initially, titles and abstracts will be screened for relevance by 2 independent reviewers. Studies that meet the inclusion criteria will proceed to full-text screening. The inclusion criteria will encompass studies focusing on HCWs’ perceptions of TB risk, research examining adherence to TB prevention and control measures, publications in English, and studies published from 2000 onwards to capture recent trends and advancements (Table S1 in Multimedia Appendix 2). Discrepancies between reviewers will be resolved through discussion or by consulting a third reviewer (Figure 2).

#### Stage 4: Charting the Data

Data extraction will be performed using a standardized charting form (Table S2 in Multimedia Appendix 2). Key information to be extracted on the characteristics of the study include the author(s), year, country, and study design; participant details, including the sample size, health care role, and setting; perceptions of TB risk; factors influencing the adherence to TB prevention measures; and main findings and conclusions. This iterative process will allow for continuous refinement of the data extraction form as necessary.

#### Stage 5: Collating, Summarizing, and Reporting Results

The extracted data will be collated and summarized to provide an overview of the existing literature. This will involve a descriptive numerical summary and a thematic analysis. The numerical summary will outline the study characteristics, while the thematic analysis will identify common themes related to HCWs’ perceptions of TB risk and factors influencing their adherence to prevention measures. This stage aims to highlight the key findings, identify gaps in the literature, and suggest implications for policy and practice.

#### Stage 6: Consultation With Stakeholders

To enhance the relevance and applicability of the findings, we will conduct consultations with stakeholders such as health care professionals, TB experts, and policymakers. These consultations will provide additional insights, validate the findings, and offer practical recommendations for improving TB prevention and control in health care settings. Stakeholder feedback will be integrated into the final report to ensure that it addresses real-world concerns and needs. By adhering to this structured and systematic methodology, this scoping review will provide a comprehensive understanding of HCWs’ perceptions of occupational TB risk and the factors influencing their adherence to preventive measures.

### Data Synthesis and Thematic Analysis

We will explore patterns in how HCWs think about the risk of occupational TB and how well they follow TB prevention and control measures by performing a thematic analysis of the qualitative and quantitative data we collected. The process will be based on Braun and Clarke’s 6-phase framework [24] for thematic analysis. The 6 steps are as follows: (1) getting to know the data, (2) making initial codes, (3) looking for themes, (4) reviewing themes, (5) defining and naming themes, and (6) writing the report. We will organize the data in order and analyze with NVivo (version 14; QSR International) to help with coding and developing themes in a systematic way. Each reviewer will code each study on their own. If they do not agree, they will talk about it or, if necessary, have a third reviewer to help. Thereafter, the codes will be arranged into groups, and then those groups will be used to develop overarching themes.

We will maintain an audit trail to make the analysis open and trustworthy, and we will hold regular team meetings to talk about new themes and make sure everyone agrees. There will be both descriptive and analytical themes in the synthesis, which will show what people think is a risky job, how they act in response to it, what helps and hurts, and how these things change in different health care settings. The last themes will be tweaked to fit the review’s goals, and the results section will show them with evidence from the studies that were used. We will consider heterogeneity across data sources by stratifying findings based on key study characteristics such as geographic region, health care setting (eg, hospital vs primary care), type of HCW, and study design (quantitative, qualitative, or mixed methods). This stratified approach will help us capture the variations in HCWs’ perceptions and adherence to TB preventive measures across different contexts.

### Ethical Considerations

As this study is a scoping review that involves the synthesis of data from publicly available literature and does not include any research involving human participants, identifiable personal data, or animals, an ethics review board assessment was not required. According to institutional and international guidelines, including the Tri-Council Policy Statement: Ethical Conduct for Research Involving Humans (article 2.2) and equivalent regional policies, research that does not involve interaction with humans or access to nonpublic identifiable data is exempt from formal ethical review. Therefore, no application was submitted to an ethics committee for this protocol.

## Results

The electronic database search was completed in June 2024, initiating the research team’s review of HCWs’ perceptions of occupational TB risk and related preventive behaviors. A total of 1345 records were identified (1234 from databases and 111 from other sources), and 667 duplicates were removed. The remaining 678 records were screened by title and abstract, with 216 progressing to full-text review. After applying eligibility criteria, 42 studies were included in the final synthesis. Screening and full-text assessments were conducted between September and October 2024. Data extraction and thematic analysis were performed in winter 2024. This review summarizes key findings, including study characteristics, participant roles and settings, risk perceptions, factors influencing adherence to TB prevention measures (such as personal protective equipment use, routine screening, and institutional support), and barriers such as stigma and poor infrastructure. At the time of submission in February 2025, the study was in the final data synthesis stage.

## Discussion

### Overview

The findings of this scoping review are expected to offer several real-world applications for health care systems, particularly in high TB burden countries. By identifying the key factors influencing HCWs’ perceptions of occupational TB risk and their adherence to preventive measures, this review will support the development of targeted, evidence-informed interventions aimed at improving workplace safety. Policymakers and hospital administrators can utilize these insights to strengthen institutional policies—such as the provision of adequate personal protective equipment, regular TB screening, and comprehensive training—while also addressing structural and psychosocial barriers, including TB-related stigma. Additionally, this review will provide a valuable foundation for researchers and TB program implementers to design context-sensitive strategies that enhance infection control efforts, protect frontline health workers, and ultimately reduce nosocomial transmission of TB. The synthesis of diverse study contexts and health care settings will further ensure that the recommendations are adaptable and applicable across a range of health system environments.

In addition, this review highlights the interaction between individual and organizational factors in influencing HCWs’ preventive actions. It is essential to consider, in addition to the resources being available, that adherence is mediated by perceptions of support from the institution in addition to the presence of a work culture that is supportive. Stigmas associated with TB, barriers to accessing human resources and workload pressures, and insufficient comprehensive training programs are other hindrances that contribute to the observed situation. The translation of these results further outlines the knowledge gap in the current literature and highlights contributory ideas to better fit potential interventions for this demographic. If these barriers are addressed through improved policies, capacity development/training initiatives, and institutional reforms, there can be a significant improvement in adherence to TB preventive measures, which could reduce the occupational risk of TB among HCWs across the world.

### Strengths and Limitations

This scoping review has some strengths. First, it has a wide scope of included study designs (qualitative, quantitative, and mixed methods) and sources that ensure a comprehensive evidence base. The addition of peer-reviewed papers and grey literature also provides a wide range of data. Second, the methodology follows the Arksey and O’Malley [14] framework, with enhancements by Levac et al [21], and guided by the Joanna Briggs Institute methodology for scoping reviews [22] to ensure rigor and transparency. Third, engagement with health care professionals, TB specialists, and policymakers increases the real-world relevance and applicability of the results. Fourth, and particularly important, by considering data from HCWs in low-, middle- and high-income countries, the results are globally relevant and add weight to a global case for policy and practice based on evidence. Finally, the review findings have potential implications for the design of targeted interventions that could enhance TB prevention and adherence among HCWs.

However, this review also had a few limitations. First, limiting the review to English language publications may miss pertinent studies published in other languages. Second, by restricting the review to studies published from 2000 onwards, earlier research and historical trends may have been overlooked. Third, using published studies may be subject to publication bias by excluding unpublished or not publicly accessible data. Fourth, differences in design, population, and setting of the included studies’ designs make data synthesis and comparison challenging. Finally, although independent reviewers strived to reduce potential bias, the interpretation of themes in this thematic analysis represented somewhat subjective accounting. These limitations should be borne in mind when interpreting the findings of the review.

### Conclusion

This scoping review is the first of its kind, to our knowledge, focusing on presenting methodological and technical guidance on assessing and understanding HCWs’ perceptions of occupational TB risk and adherence to preventive measures. This scoping review will systematically map the existing literature to address critical knowledge gaps and provide a comprehensive overview of the factors influencing HCWs’ behaviors across different health care settings. These findings will guide policymakers, health care institutions, and researchers in formulating targeted strategies and interventions to improve TB prevention and control efforts, particularly in high-burden areas. The findings from this review will support upcoming research and operationalization to be implemented to ensure the safety and wellness of the primary receptors of frontline risk, that is, humanitarian health workers, across the globe.

## Appendix

Multimedia Appendix 1 PRISMA-ScR checklist.

Multimedia Appendix 2 Study selection and data extraction.

## Abbreviations

HCW: health care worker
LMICs: low- and middle-income countries
PRISMA-ScR: Preferred Reporting Items for Systematic reviews and Meta-Analyses extension for Scoping Reviews
TB: tuberculosis
WHO: World Health Organization
