# Supplementary material for: Perceptions of Occupational Risk and Adherence to Tuberculosis Prevention Among Health Care Workers: Protocol for a Scoping Review
Source: JMIR Res Protoc. 2025 Sep 22;14:e64037. doi: 10.2196/64037 (PMC12501531; doi:10.2196/64037)
Supplement: Multimedia Appendix 2 [file resprot_v14i1e64037_app2.docx]

**Multimedia Appendix 1**

**Table S1.** Inclusion and exclusion criteria form.

| Criteria | | Review result |
| --- | --- | --- |
| **Inclusion** | | |
|  | Health care workers (HCWs), including doctors, nurses, laboratory technicians, and support staff directly involved in patient care.  HCWs currently employed in health care settings such as hospitals, clinics, or health centers.  A minimum of six months of experience in a health care setting.  Studies focusing on health care workers’ perceptions of occupational risk related to tuberculosis (TB).  Studies examining adherence to TB prevention and control measures.  Any health care facilities, including hospitals, primary care, or clinics.  Studies conducted in any country and health care setting.  All research designs: observational studies, randomized controlled trials, systematic reviews, case studies, qualitative studies, and clinical guidelines.  Publications in English, from the year 2000 onwards | [] Yes [] No |
|  | Used or proposed quality indicators for HCW’s perception | [] Yes [] No |
| **Exclusion** | | |
|  | Studies focusing solely on populations such as pregnant women or juveniles without specific reference to HCWs. | [] Yes [] No |
|  | Publication in the form of editorials, letters to the editor, comments, case reports or narrative case reports | [] Yes [] No |

**Table S2.** Extraction data form.

| Data | | Response |
| --- | --- | --- |
| Author | |  |
| Year of publication | |  |
| Research location | |  |
| Design | |  |
| Objective | |  |
| Type of sources | |  |
| **List of indicators** | | |
|  | Indicator name |  |
|  | Description of indicators (numerator and denominator if any) |  |
|  | Indicator objectives |  |
|  | Setting of health care facilities | [] Hospital, [] Primary care, [] Clinic |
|  | Type of indicators | [] Input, [] Process, [] Output |
|  | Level of indicators | [] Patients, [] Institutions, [] Health systems |
|  | Quality domain |  |
|  | Validity test | [] Done, [] None |
| Author recommendations | |  |
| Reviewer’s note | |  |
